# Assessment of clinical utility and predictive potential of pre-chemotherapy soluble urokinase plasminogen activator receptor: Observational single center study

**DOI:** 10.17305/bjbms.2022.7857

**Published:** 2023-03-16

**Authors:** Elina Beleva, Snezhana Stoencheva, Tanya Deneva, Ivanka Nenova, Zhanet Grudeva-Popova

**Affiliations:** 1 Department of Clinical Oncology, Medical Faculty, Medical University of Plovdiv, Plovdiv, Bulgaria; 2 University Hospital “Sveti Georgi” EAD, Plovdiv, Bulgaria; 3 Department of Clinical Laboratory, Faculty of Pharmacy, Medical University of Plovdiv, Plovdiv, Bulgaria

**Keywords:** Cancer, biomarkers, plasminogen activator receptor, urokinase type, progression-free survival, hemostasis

## Abstract

Alteration of urokinase plasminogen activator receptor (uPAR) in neoplasms is a pre-requisite for invasiveness and metastatic ability. In the present study, we aimed to evaluate the relationship of pre-chemotherapy soluble uPAR (suPAR) with the odds for metastasis, lack of disease control, and its predictive ability for progression-free survival (PFS). Baseline plasma suPAR levels were measured by ELISA in 89 patients with various cancers prior to initiation of systemic treatment. Patients were followed prospectively until metastatic progression or death. TCGA Pan-Cancer dataset was mined for available RNAseq expression data of the *PLAUR* gene in patients with breast, colon, and lung cancer, and the relevant genomic and clinical data were extracted for further analysis. Pre-chemotherapy suPAR levels were significantly associated with white blood cell counts and fibrinogen and were significantly elevated both in patients with metastatic disease and in patients with progression. Increasing suPAR was significantly associated with odds for progression in the prespecified multivariate analysis (odds ratio 2.47, 95% confidence interval 1.3–5.11). In univariate Cox regression, suPAR was predictive of shortened PFS (hazard ratio 1.065, 95% confidence interval 1.002–1.13; *p* = 0.041). There was a trend toward shortened PFS in patients with higher baseline suPAR levels (cutoff 8.1 ng/mL). In the TCGA lung cancer cohort, *PLAUR* mRNA expression was significantly associated with shortened PFS in both univariate and multivariate analyses. High *PLAUR* gene expression conferred significant survival disadvantage only in patients with colon and lung cancer. SuPAR may bear predictive potential for adverse outcomes in cancer, but its utility as a biomarker seems to be more pronounced in cancers with associated inflammatory state.

## Introduction

Interaction between fibrinolytic system and cancer has been recognized ever since the observation that increased fibrinolytic activity of neoplastic cells promotes tumor invasiveness [1]. This has mainly been attributed to deregulation of the urokinase plasminogen activator (uPA), urokinase plasminogen activator receptor (uPAR), and plasminogen activator inhibitor-1 (PAI-1) axis [2]. As far as the physiologic role of uPA/uPAR/PAI-1 system is to tightly control the processes of extracellular matrix degradation, cellular migration, wound healing, and tissue remodeling, alteration of its activity in neoplastic state is a pre-requisite for invasive phenotype and ability to metastasize. Furthermore, uPA and PAI-1 have been validated as markers for tumor invasiveness in breast cancer and their expression levels are used for treatment decision making in early stage breast cancer [3]. Among the different fibrinolytic components, uPAR has also been established as an important mediator of tumor proliferation, adhesion, chemotaxis, migration, and angiogenesis. Its overexpression in cancerous tissue is almost unequivocal, while sparse or low expression is seen in adjacent normal or homeostatic tissues [4]. Despite the substantial evidence on the clinical relevance and prognostic significance of tissue-expressed uPAR as a biomarker for cancer progression, its use is limited due to the requirement for tissue specimen availability [5, 6]. The soluble form (suPAR), released upon proteolytic cleavage, represents a promising surrogate candidate biomarker over uPAR [7]. Release of suPAR is triggered by inflammatory stimuli and immune activation. Thus, it has been proposed as suitable biomarker for systemic chronic inflammation [8]. Elevated levels of suPAR have been found in various cancers and increasing evidence suggests that suPAR may hold predictive ability for treatment response and mortality in cancer patients [9, 10]. However, despite increasing evidence of its clinical utility as a cancer biomarker, suPAR has not yet found a place in the clinical pathway of cancer patients. It is still unclear in what biomarker context suPAR can be used, and establishing an association with specific outcomes in specific clinical populations is still required for the use of suPAR’s diagnostic capability [8].

**Table 1:** Study REMARK profile

***A) Patients, treatment, and variables***			
**Study and marker**		**Remarks**		
Marker (continuous or categorical)		suPAR (ng/mL) = soluble urokinase plasminogen activator receptor (continuous)		
		suPAR*√* = square root transformed suPAR (ng/mL) to remove positive skewness		
Further variables (variables collected, variables available for analysis, baseline variables, patient and tumor variables)		*Continuous:* v1 = age, v8 = fibrinogen, v9 = WBC; v10 = BMI; v11 = MPV/PLT ratio;		
		*Remarks:* Continuous data are presented as mean + standard deviation or median (range) as appropriate.		
		*Categorical:* v2 = sex (female, male), v3 = smoking history (no, yes), v4 = diagnosis (breast, lung, ovary, colon), v5 = surgical resection (totally resected, residual tumor), v6 = metastatic disease: no (I–III stage), yes (IV stage), v7 = tumor response: controlled disease (stable disease, partial remission, complete remission), progression.		
Inclusion criteria		Age > 18 years, histologically proven diagnosis of breast, lung, ovary, or colorectal cancer, any disease stage, newly diagnosed chemotherapy-naïve patients or patients indicated for first line of chemotherapy after one prior regimen and documented progression after at least 6 months disease free-interval.		
Exclusion criteria		History of arterial or venous thrombosis within 3 months prior to study inclusion, cardio-vascular co-morbidities: heart failure NYHA class III/IV, arterial by-pass surgery, angioplasty and vascular stenting, valve prosthesis, performance status on the ECOG scale > 4, oral contraceptive use, known hereditary prothrombotic polymorphisms, acute viral or bacterial infection 2 weeks prior to study inclusion, exacerbation of chronic inflammatory condition 2 weeks prior to study inclusion, anticoagulant treatment with vitamin K antagonists and/or direct oral anticoagulants within last 3 months, treatment of vitamin K within one month prior to study inclusion.		
**Patients**		***n***	**Remarks**	
Assessed for eligibility		102	Patients underwent staging procedures and treatment evaluation according to the National Standards of the Bulgarian Oncology Society.	
Excluded		13	General exclusion criteria (n=5), missing data on suPAR (n = 8)	
Included		89	Newly diagnosed (n = 60) or first line after documented progression with > 6 months documented disease-free interval (n = 29)	
With outcome event		83	PFS: distant metastasis or death. Remarks: 6 patients excluded (3 did not undergo chemotherapy, 3 were lost to follow-up).	
***B) Statistical analyses of survival outcomes***			
**Aim**	**Patients, *n***	**Events**	**Variables considered**	**Results/remarks**
A1: Univariate	83	26	suPAR	Table 4, PFS: significant
A2: Multivariate	83	26	suPAR, v4	Table 4, Fig. 4A, PFS: not significant

suPAR: Soluble urokinase plasminogen activator receptor; WBC: White blood cells; BMI: Body mass index; MPV/PLT: Mean platelet volume/platelet count; NYHA: New York Heart Association; ECOG: Eastern Cooperative Oncology Group; PFS: Progression-free survival; A1: Analysis 1; A2: Analysis 2; v: Variable.

In the current study, we measured suPAR levels in cancer patients prior to initiation of systemic cancer treatment and followed them until metastatic progression or death. We also validated observed associations from the clinical cohort on *PLAUR* RNAseq expression data from publicly available TCGA dataset. We hypothesized that suPAR levels correlate with inflammatory markers, differ between tumor sites due to the biologically inherent differences in tumor aggressiveness, and that suPAR levels are lower in patients with totally resected tumors. Further, we supposed that higher levels of suPAR may be predictive of metastatic disease at presentation, progressive disease, and shortened progression-free survival (PFS).

## Materials and methods

### Patients and study design

A prospective cohort of 102 cancer patients was recruited between April 2013 and April 2014 at the Clinic of Medical Oncology, University Hospital “Sveti Georgi” EAD, Plovdiv, Bulgaria, from all patients referred to the clinic for initial evaluation, staging, and initiation of systemic cancer treatment. Inclusion and exclusion criteria are presented in the REMARK profile of study (Table 1). Prior to initiation of any systemic treatment (~four weeks after any cancer-related surgical procedure), blood was sampled for the analysis of suPAR, complete blood count, coagulation times, and fibrinogen. All patients were examined at 3–6 months intervals and followed up until a relevant clinical endpoint was met or the study ended (April 2015). As primary clinical endpoint we examined PFS, defined as the time between treatment initiation and distant metastatic occurrence or death. The rationale for choosing PFS as the primary endpoint was that cell membrane-bound uPAR is a critical factor for invasion and metastasis in cancer [6]. As secondary outcomes of interest, we considered metastatic disease at presentation and progression defined as an event requiring a new line of treatment: biochemical progression (as defined by RECIST), local, loco-regional relapse, or occurrence of distant metastases, whichever comes first. Tumor response assessment was performed according to national standards and RECIST [11, 12]. The study is presented in accordance with REMARK guidelines [13].

### Specimen characteristics and assay methods

Peripheral blood was collected by atraumatic venipuncture of the cubital vein in EDTA-K_3_ tubes (Monovette Potassium EDTA, 2.7 ml) for suPAR determination. Samples were centrifuged at 3000*g* for 10 min after which plasma supernatant was removed. Plasma suPAR concentration was determined by an enzyme-linked immunosorbent assay (suPARnostic Standard ELISA, ViroGates, Denmark), which detects both full-length (D1D2D3) and cleaved (D2D3) suPAR; concentrations are expressed in ng/mL. Coagulation times were measured on a Sysmex CS 2000 coagulometer (Siemens diagnostica), fibrinogen according to Clauss, complete blood count on ADVIA 2120i analyzer (Sysmex diagnostic) [14].

### In silico validation and analysis of *PLAUR* gene expression

TCGA Pan-Cancer dataset (https://www.cancer.gov/tcga) was accessed via UCSC Xena (https://xenabrowser.net/), which is an integrative platform aiding functional genomic data analysis for clinical research [15, 16]. Patients with breast, colon, and lung cancer whose primary tumors had available RNAseq expression data for the gene encoding uPAR—*PLAUR* were selected. There were no ovary cancer cases with available matching data. Additionally, we extracted data on following variables with relevance to our clinical cohort analysis: RNAseq expression data on fibrinogen chain genes—*FGA*, *FGB*, *FGG*, age, gender, progression-free interval events and progression-free interval time in days. No data on smoking history, residual tumor, and clinical stage were available for selected cases. Dataset consisted of 2566 samples.

### Ethical statement

The study was approved by the Ethics Committee of the Medical University of Plovdiv, Bulgaria (P-4012/28.10.2013) and complies with the Declaration of Helsinki. All study subjects provided written informed consent prior to any study-related procedures. Subject-related data were captured on individual hard copies for each subject.

### Statistical analysis

Definition of variables is presented in Table 1. All statistical analyses were performed in R programming language v.4.1.3 and RStudio with packages *ggpubr*, *caret*, *car*, *ggplot, VGAM, cowplot, ggsci*, *fmsb*, *ROCit*, *survival*, *survminer*, and *memisc* [17–31]. Welch’s t-test was used to compare two groups. Differences across cancer types were assessed by ANOVA with an interaction term between diagnosis and gender. Predictive ability of suPAR for metastatic and progressive disease was evaluated by univariate and multivariate logistic regression. Discriminatory ability of suPAR for the secondary endpoints was assessed by ROC analysis. Association with PFS was analyzed by univariate and multivariate Cox regression and survival differences calculated by Kaplan–Meier product limit method. Cutoff values for patient dichotomization were identified based on Youden index criterion in ROC analysis for binary classification of a PFS event. All multivariate models were pre-specified with the inclusion of diagnosis to account for tumor-inherent differences in biological heterogeneity as it pertains to the clinical outcomes. Additionally, post-hoc analysis was performed with the inclusion of gender and smoking history in the multivariate models. The study was not designed to detect a specified effect size. Missing data were handled by complete case analysis. A *p*-value <0.05 was considered the threshold for statistical significance. Programming code and associated data are available from the authors upon reasonable request.

## Results

**Figure 1.**
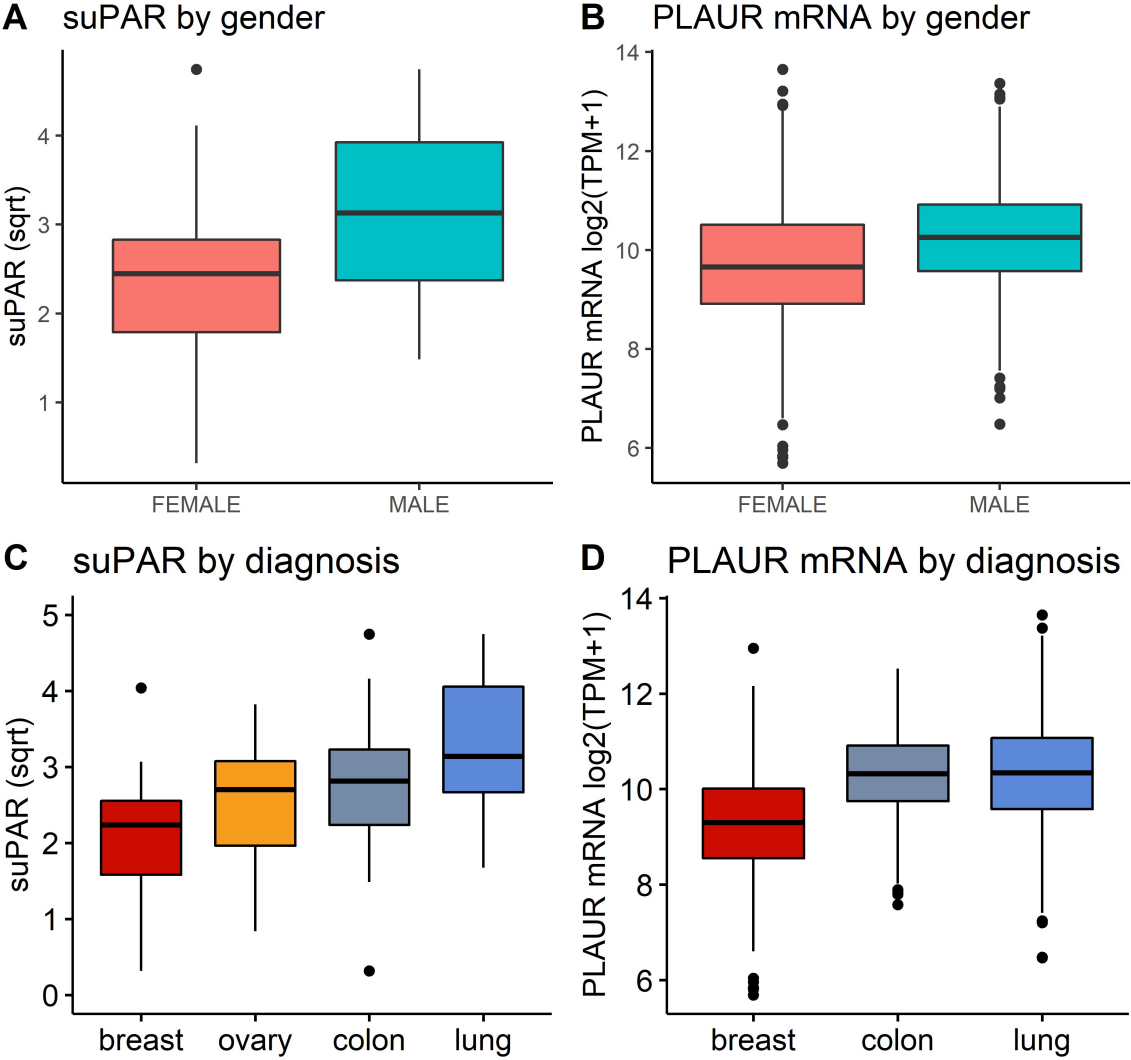
Differences in (A) suPAR levels and (B) *PLAUR* mRNA expression by gender; (C) Plasma suPAR levels compared across cancer types; (D) *PLAUR* mRNA expression compared across samples from colon, lung, and breast cancer primary tumors. suPAR: Soluble urokinase plasminogen activator receptor.

REMARK profile of the study with number of patients at each stage and reasons for non-participation are presented in Table 1 and Table S1. Patients’ characteristics and baseline pre-chemotherapy laboratory parameters are presented in Table 2. Mean suPAR ng/mL was 7.86 (+5.28) and mean suPAR*√* was 2.549 (+0.975). Significantly higher suPAR*√* was observed in male than in female patients (3.17 ± 0.891 vs 2.33 ± 0.893; *p* = 0.00006) (Figure 1A). Elevated suPAR*√* was also observed in patients with positive smoking history compared to non-smokers (3.29 ± 0.87 vs 2.36 ± 0.88; *t*(47.19)= −4.36; *p* = 0.00003). Significant weak correlations were found with white blood cell (WBC) count and fibrinogen (fbg), which were confirmed as significant predictors by linear regression (F(1, 83) = 8.089; *p* = 0.005**_fbg_** and F(1, 88) = 8.617; *p* = 0.004**_WBC_**). The regression equations were: predicted suPAR*√* = 1.764 + 0.223 × fbg (g/L); and predicted suPAR*√* = 1.73 + 0.099 × WBC (g/L). No correlation was detected with age, body mass index, and mean platelet volume/platelet count (MPV/PLT) ratio (Table S2). No significant difference was found between patients with residual tumors and those with completely resected tumours. SuPAR*√* values were significantly different across primary tumor sites (F (3, 82) = 7.449; *p* = 0.0001). Mean values increased from breast (2.09 + 0.839) to ovary (2.54 + 0.763), colon (2.77 + 1.04) and lung cancer (3.27 + 0.86) (Figure 1). Tukey *post-hoc* test also revealed significant interaction effect between tumor site and gender. In TCGA validation cohort, *PLAUR* was upregulated in male patients and significantly correlated with age (Figure 1B, Table S1). *PLAUR* expression correlated significantly with *FGA*, *FGB*, and *FGG* expression (Table S1). There was significant upregulation of *PLAUR* in lung and colon cancer compared to breast cancer (Figure 1). The ANOVA revealed significant main effects for both gender and diagnosis, but no interaction was observed.

**Table 2:** Characteristics of study subjects

	**Number**	**%**
Patients enrolled	89	100
Characteristic		
*Age, years*		
Median	60	
Range	21–81	
*Sex, n*		
Female	57	64
Male	32	36
*BMI, kg/m^2^*		
Median	24.77	
Range	16.16–36	
*Smoking history, n*		
No	63	71
Yes	26	29
*Surgery, n*		
Inoperable	13	15
Partial resection	5	5
Total resection	71	80
*Diagnosis, n*		
Breast	28	31
Lung	20	23
Ovary	15	17
Colon	26	29
*Stage, n*		
I	14	16
II	30	34
III	27	30
IV	18	20
*Chemotherapy, n*		
Adjuvant	60	67
1^st^ line	29	33
*Progression, n*		
Yes	32	36
No	57	64
*PFS event, n*		
Yes	26	31
No	57	69
*Follow-up time, days*		
Median	214	
Range	9–583	
*MPV/PLT ratio*		
Median	0.025	88
Range	0.007–0.033	
*suPAR ng/mL*		
Median	6.5	100
Range	0.0–22.5	
*suPAR*√**		
Median	2.549	100
Range	0.32–2.63	

suPAR: Soluble urokinase plasminogen activator receptor; suPAR*√*: Square root transformed suPAR (ng/mL) to remove positive skewness; BMI: Body mass index; MPV/PLT: Mean platelet volume/platelet count; PFS: Progression-free survival.

**Figure 2.**
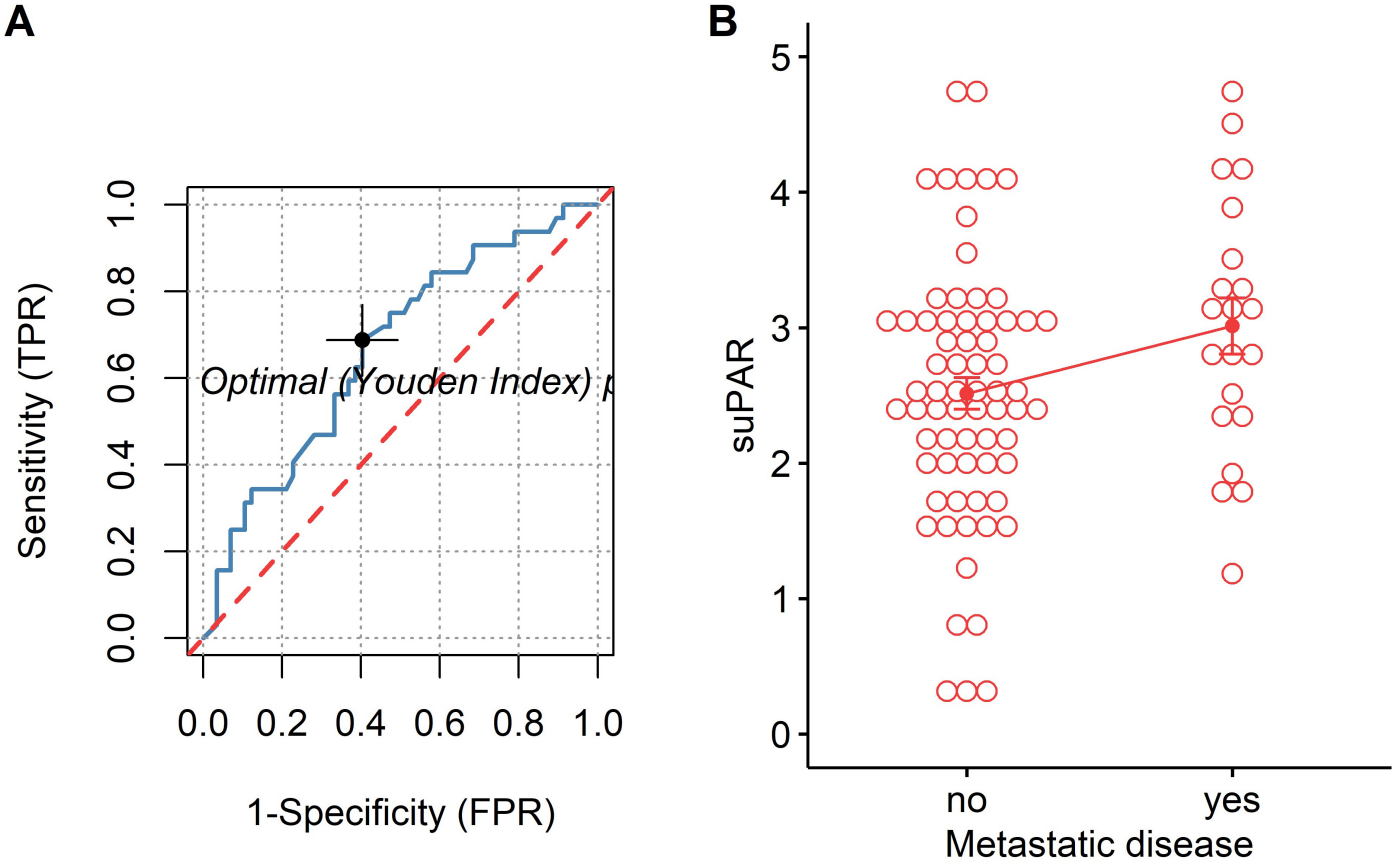
(A) ROC curve of suPAR for the presence of distant metastasis, cutoff 2.75 (empirical line – blue, chance line – red) and (B) Patients with metastatic disease (3.01 + 0.947) vs patients without (2.51 + 0.96), *t* (33.7) = −2.10, *p* = 0.043. TPR: True positive rate; FPR: False positive rate; suPAR: Soluble urokinase plasminogen activator receptor; ROC: Receiver operating characteristics.

**Figure 3.**
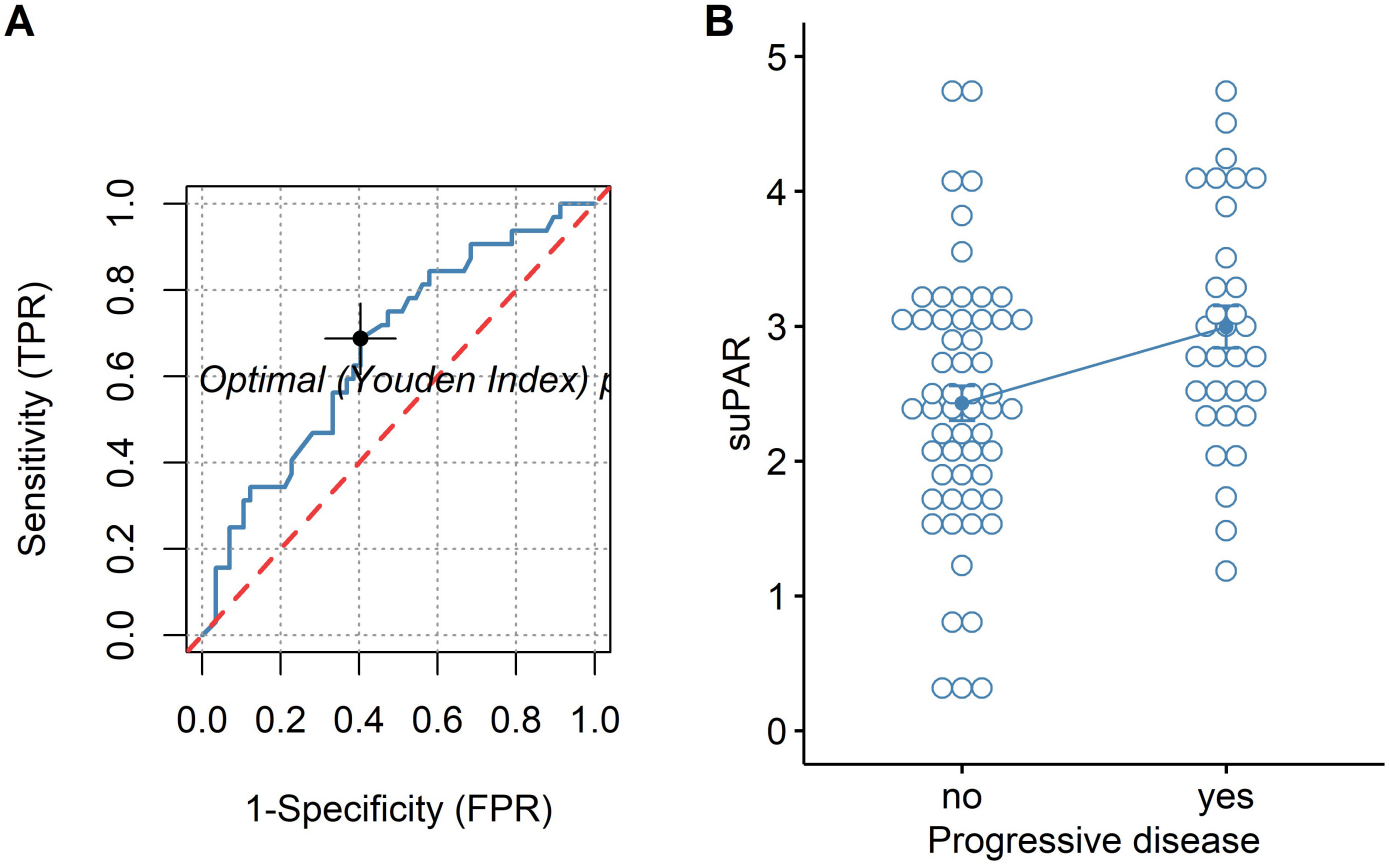
(A) ROC curve of suPAR for disease progression (empirical line – blue, chance line – red) and (B) Patients with progressive disease (2.993 + 0.885) vs disease control (2.427 + 0.971), cutoff value 2.549, *t* (33.7) = −2.10, *p* = 0.043. TPR: True positive rate; FPR: False positive rate; suPAR: Soluble urokinase plasminogen activator receptor; ROC: Receiver operating characteristics.

**Figure 4.**
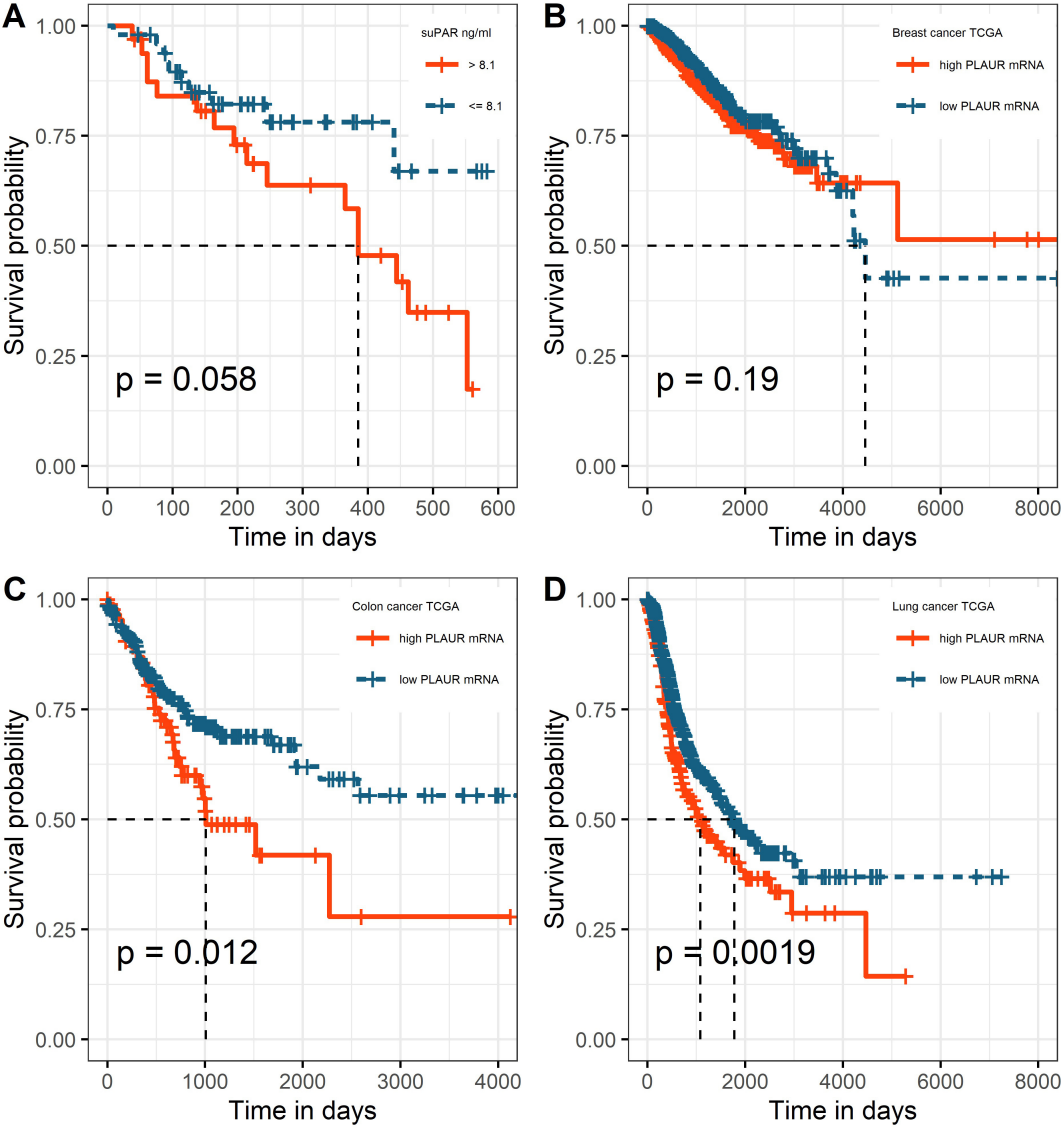
Kaplan-Meier survival curves with respect to the threshold for binary classification of PFS event: (A) high vs low suPAR (cut-off 8.1 ng/mL); high vs low *PLAUR* gene expression in (B) breast cancer TCGA (cutoff 9.35 log2(TPM+1)), (C) colon cancer TCGA (cutoff 10.99 log2(TPM+1)), and (D) lung cancer TCGA (cutoff 11.05 log2(TPM+1)). suPAR: Soluble urokinase plasminogen activator receptor; PFS: Progression-free survival.

For both univariate and multivariate models and either outcome measurement, there was linearity between the logit transformation of the dependent variables and the continuous predictor suPAR*√*. Multicollinearity was not detected. Influential data points as identified by Cook’s distance measurements were replaced with the median value of suPAR*√* for each model respectively. Baseline suPAR*√* values were significantly elevated in patients with metastatic disease and/or progression compared to patients without distant metastasis or disease control (Figures 2B and 3B). ROC analysis determined an area under the curve (AUC) of 0.649 (95%CI 0.509–0.79) for suPAR*√* discriminatory ability for metastasis and showed sensitivity of 66% and specificity of 62% of suPAR*√* at the Youden index point (Figure 2A). SuPAR*√* was found to be significantly predictive of increased odds for presence of distant metastasis only in univariate logistic regression (Table 3A). Regarding its ability to discriminate between patients whose disease would subsequently progress and those whose would not, suPAR*√* had an AUC value of 0.665 (95%CI 0.548–0.78) and showed sensitivity of 66% and specificity of 60% (Figure 3A). SuPAR*√* was found to be significantly predictive of increased odds for progression in both univariate and prespecified multivariate analysis (Table 3B). In the post-hoc multivariate model with correction for gender and smoking history, suPAR*√* did not retain significance as a predictor for disease progression, albeit higher levels were associated with increasing odds (Table 3B). Pre-chemotherapy suPAR*√* was found to be significantly predictive of shortened PFS time in univariate Cox regression analysis (Table 4A). In the multivariate Cox analysis, increasing suPAR*√* levels were still associated with shortened PFS time, but association was no longer significant (Table 4A). The median PFS in patients with high suPAR*√* was 385 days (95%CI 254–not estimable), while patients with low suPAR*√* did not reach median PFS during the observation period (median not estimable, 95% CI 440–not estimable). Difference in the survival distributions between low and high groups approximated significance (Figure 4A). Upregulation of *PLAUR* mRNA expression was significantly associated with shortened PFS only in lung cancer (Table 4D). Significant survival differences between patients with high and low *PLAUR* expression were observed for patients with lung and colon cancer (Figure 4B–4D).

**Table 3:** Univariate and multivariate: logistic regression for the prediction of (A) metastatic disease and (B) tumor response by suPAR*√* in the clinical cohort

***A) Metastatic Disease, n = 89***			
	**Univariate^suPAR^**		**Multivariate**			
			**Prespecified**		***Post-hoc***	
	**OR (95% CI)**	***p***	**OR (95% CI)**	***p***	**OR (95% CI)**	***p***
suPAR*√*	2.43 (1.24–5.19)	0.01*	1.53 (0.80–3.07)	0.208	1.36 (0.67–2.87)	0.39
Diagnosis						
Breast			Ref.		Ref.	
Lung			4.35 (0.90–25.5)	0.077	2.28 (0.31–17.12)	0.41
Ovary			3.52 (0.71–20.36)	0.13	5.16 (1.00–31.5)	0.055
Colon			1.17 (0.218–6.82)	0.84	0.567 (0.072–4.07)	0.56
Gender						
Female					Ref.	
Male					5.26 (1.06–33.98)	0.33
Smoking						
No					Ref.	
Yes					0.52 (0.12–1.88)	0.33
***B) Tumor response, n = 89***			
	**Univariate^suPAR^**		**Multivariate**			
			**Prespecified**		**Post-hoc**	
	**OR (95% CI)**	***p***	**OR (95% CI)**	***p***	**OR (95% CI)**	***p***
suPAR*√*	3.02 (1.66-6.13)	0.0008***	2.47 (1.3–5.11)	0.0084**	1.66 (0.89–3.22)	0.11
Diagnosis						
Breast			Ref.		Ref.	
Lung			3.39 (0.85–14.4)	0.086	3.30 (0.66–17.64)	0.14
Ovary			1.49 (0.35–6.2)	0.57	1.55 (0.36–6.46)	0.53
Colon			0.35 (0.07–1.47)	0.16	0.41 (0.07–1.91)	0.27
Gender						
Female					Ref.	
Male					1.13 (0.25–4.99)	0.86
Smoking						
No					Ref.	
Yes					1.36 (0.38–4.79)	0.62

Groups: A) Metastatic disease: no vs yes; B) Tumor response: disease control vs progression. OR > 1 favors A) distant metastasis and B) progression; *** < 0.001 < ** < 0.01 < * < 0.05. suPAR: Soluble urokinase plasminogen activator; suPAR*√*: Square root transformed suPAR (ng/mL) to remove positive skewness; OR: Odds ratio; CI: Confidence interval.

**Table 4:** Cox regression for prediction of PFS by suPAR ng/mL and by *PLAUR* mRNA log2(TPM+1) within cancer type

***A) suPAR ng/mL, n = 83***			
	**Univariate^suPAR^**		**Multivariate**			
			**Prespecified**		**Post-hoc**	
	**HR (95% CI)**	***p***	**HR (95% CI)**	***p***	**HR (95% CI)**	***p***
suPAR	1.065 (1.002–5.13)	0.041*	1.02 (0.947–1.104)	0.57	1.36 (0.67–2.87)	0.39
Diagnosis						
Breast			Ref.		Ref.	
Lung			3.384 (0.998–11.47)	0.0503	2.28 (0.31–17.12)	0.41
Ovary			1.38 (0.36–5.2)	0.63	5.16 (1.00–31.5)	0.055
Colon			1.007 (0.25–4.067)	0.99	0.567 (0.072–4.07)	0.56
Gender						
Female					Ref.	
Male					5.26 (1.06–33.98)	0.33
Smoking						
No					Ref.	
Yes					0.52 (0.12–1.88)	0.33
***B-D) PLAUR mRNA log2(TPM+1)***			
	**Univariate**			**Post-hoc multivariate**		
	**HR (95% CI)**		***p***	**HR (95% CI)**		***p***
**B) TCGA Breast Cancer, *n* = 1097**			
_PLAUR_mRNA	1.055 (0.90–1.23)		0.5	1.054 (0.899–1.235)		0.51
Gender^¶^						
Female				Ref.		
Male				0.699 (0.097–5.00)		0.72
**C) TCGA Colon Cancer, *n* = 452**			
_PLAUR_mRNA	1.087 (0.87–1.36)		0.46	1.094 (0.87–1.36)		0.42
Gender						
Female				Ref.		
Male				1.237 (0.86–1.78)		0.25
**D) TCGA Lung Cancer, **n** = 1017**			
_PLAUR_mRNA	1.18 (1.072–1.30)		0.000751***	1.18 (1.071–1.30)		0.00078***
Gender						
Female				Ref.		
Male				1.00 (0.81–1.238)		0.99

HR < 1 favors decreasing suPAR ng/mL and lower *PLAUR* mRNA expression log2(TPM+1)**.** *** < 0.001; ** < 0.01; * < 0.05; ^¶^There were 12 male breast cancer patients in the dataset. suPAR: Soluble urokinase plasminogen activator; suPAR*√*: Square root transformed suPAR (ng/mL) to remove positive skewness; HR: Hazard ratio; CI: Confidence interval.

## Discussion

In the present study, we evaluated prognostic significance of the soluble isoform of fibrinolytic receptor uPAR as a biomarker for adverse outcomes in cancer patients. Due to the small number of participants in our clinical cohort, we additionally analyzed RNAseq data of *PLAUR* gene expression in an independent cohort of cancer patients as in silico validation strategy.

Given that suPAR has been proposed as a marker of systemic chronic inflammation in general population, we analyzed its relationship with inflammatory state in cancer patients. Elevation of suPAR was significantly related to increasing fibrinogen and WBC values in our study but not to other factors associated with inflammation, such as age, body mass index, and MPV/PLT ratio. Additionally, *PLAUR* gene expression correlated significantly with the expression of genes encoding fibrinogen chains. Direct relationship between suPAR and inflammatory biomarkers could be expected since immune cells are major source for uPAR. Its expression on cellular surface is upregulated upon immune activation during wound healing, tissue remodeling, injury, and in cancer tissues. Moreover, transcriptional factors involved in inflammatory pathways as well as in the regulation of cellular differentiation, migration, and apoptosis are known to regulate the basal expression of uPAR. Overall, translational studies suggest that inflammatory signaling pathways are responsible for upregulation of uPAR [7]. This potentially places suPAR at the intersection between hemostatic system and inflammation and supports the notion that hemostatic factors promote cancer pathogenesis through modulation of inflammatory host response.

Confirming our initial assumption that suPAR may differ across different tumor sites reflecting inherent biologic differences in aggressiveness and invasive potential, we found that patients with lung cancer have highest levels in the studied cohort, also supported by finding of *PLAUR* upregulation in lung and colorectal cancer compared to breast cancer. This difference was significant for the main effect of diagnosis in both datasets. Observed higher suPAR levels as well as *PLAUR* expression in male patients seems to be relative to the tumor type. Importantly, patients with smoking history in our study had significantly elevated suPAR*√* values and male gender was predominantly observed in that group (as assessed by chi-square test, data not shown). Smoking is an established lifestyle risk factor strongly associated with higher suPAR levels in general population and smoking cessation was shown to lower plasma suPAR in a randomized controlled study [32, 33]. Moreover, *in vitro* studies on normal human bronchial epithelial cells have revealed that cigarette smoke induces expression of *PLAUR* splice variant with modified terminal exon which translates into alternate soluble isoform lacking the glycosylphosphatidylinositol anchor [34]. Therefore, association of lung cancer with smoking exposure may partly explain findings of highest suPAR*√* levels and *PLAUR* mRNA expression in lung cancer patients. Noteworthy, tumor site specific hemostatic perturbations have also long been recognized. Lung cancer is associated with remarkably high thrombotic tendency and more prominent hemostatic alterations, while breast cancer confers one of the lowest risks of hemostatic perturbations among cancer diagnoses [35]. Thus, suPAR significance as a biomarker in cancer may be contextually dependent on the tumor type and associated lifestyle risk factors.

Given that uPAR is a key factor to confer metastatic potential to tumors, we tested the hypothesis whether its soluble isoform holds an ability to predict tumor’s likely propensity to metastasize. In this study, significantly higher suPAR levels were found in stage IV disease and increasing suPAR levels were associated with greater odds for distant metastasis at presentation in univariate analysis. Similarly, other studies indicate that elevation of suPAR levels depends on the extent of metastasis [36–38]. On the contrary, when comparing suPAR levels between different clinical stages in prostate and ovarian cancer, no differences were found [39, 40]. In addition, ROC analysis in our study showed that the ability of suPAR to detect the presence of metastases was not satisfactory: the estimated AUC was 0.65, with the lower limit at the chance line. As data on the association of suPAR with metastasis seem contradictory, its usefulness as a biomarker for predicting metastatic disease requires further confirmation in a larger patient cohort and in selected tumor types.

Finally, we analyzed suPAR performance in predicting treatment response and unfavorable disease outcome. Our results indicate that patients who do not achieve disease control have higher pre-chemotherapy suPAR values. In addition, baseline suPAR significantly predicted progressive disease in multivariate analysis and was found to be associated with higher risk for metastasis or death in univariate Cox regression. However, correction for gender and smoking history abrogated significance level for prediction of progressive disease. In TCGA cohort, association of *PLAUR* gene expression with shortened PFS was found only in patients with lung cancer. This association remained significant even when corrected for gender in the post-hoc model, with the stipulation that no correction for smoking could be entailed for this dataset. Few studies have evaluated suPAR association with PFS. In a biomarker study of metastatic colorectal cancer patients, pre-chemotherapy suPAR levels were identified as significant predictor of decreased overall survival, but not of PFS [41]. On the contrary, in another cohort of metastatic colorectal cancer patients, suPAR levels were significantly associated with shortened PFS in univariate analysis [42]. In patients receiving checkpoint inhibitor therapy for various cancers, baseline suPAR levels were significantly associated with survival, defined as the time from treatment initiation to death. Taken together, this data suggest that suPAR holds potential for predicting adverse outcomes in cancer. However, only in one of these studies, smoking was assessed as patient baseline characteristic, but without being considered as confounding factor in the predictive models. Because smoking and smoking cessation can alter suPAR levels in the general population, as already mentioned, it would be feasible to stratify patients by smoking status in future studies assessing suPAR as a prognostic marker in cancer. In this study, we observed congruent results between suPAR*√* and *PLAUR* mRNA with respect to factor effects of gender and diagnosis. However, TCGA subgroup survival analysis has demonstrated that *PLAUR* mRNA expression significantly predicts PFS only in lung cancer patients, which potentially alludes to suPAR bearing predictive ability for PFS specifically in this cancer type. Still, inferences on the better prognostic potential of suPAR in lung cancer compared to breast and colon cancer may not be as straightforward, because discrepancies between tissue mRNA expression and suPAR antigen could be expected. In a study comparing primary tumor *PLAUR* mRNA expression and suPAR levels as predictors of prognosis and survival in prostate cancer, suPAR, but not tissue mRNA, correlated significantly with overall survival [43]. As suggested by the previous studies, cellular uPAR levels may not strictly correlate with mRNA levels because important intermediaries as uPA can stimulate uPAR tissue overexpression. In addition, it has been shown that wild-type and exon 4/5-deleted *PLAUR* mRNA transcript variants are predictive of disease metastasis free survival and overall survival in breast cancer patients, but do not hold predictive significance for PFS and overall survival in ovarian cancer patients [44]. Therefore, presence of alternatively spliced *PLAUR* mRNA isoforms in cancer tissue and their potential impact on survival should be accounted for as they may also contribute to discrepant results on prognostic utility of uPAR antigen or *PLAUR* mRNA between cancer types.

We consider the heterogenous patient population a major limitation of our study. We tried to address this by incorporating diagnosis into the multivariate models and performing in silico analysis on TCGA cohort. Further, small sample size and consequently small number of outcome events are other limitations, restricting post-hoc subgroup analyses in the clinical cohort.

## Conclusion

In this study, we estimated of the diagnostic utility of suPAR for discriminating presence of distant metastasis at presentation and subsequent progression. This may be useful for the design of future diagnostic accuracy study of suPAR when defining the target region in the ROC space and minimally acceptable criteria for predefined performance [44]. Its utility as a biomarker seems to be more pronounced in cancers with associated inflammatory state, such as lung cancer, as it rather reflects general systemic response of the neoplastic state; however, the context of prior exposure to suPAR modifying factors should be accounted for. This places suPAR at the forefront of potential biomarkers of systemic chronic inflammation in cancer. Given that suPAR levels show predictive potential for progressive disease and PFS, we propose that the most suitable role for suPAR in the clinical pathway of cancer patients would be as an add-on test after initial work-up and staging in patients with early-stage inflammation-driven cancers. Still, findings from this study need to be further assessed and confirmed in a specifically designed diagnostic accuracy study with larger patient sample size and within homogenous patient population.

## Supplemental Data

**Table S1:** Distribution of patients by histology types and cancer diagnosis

**Cancer diagnosis**	**Histology type**	**Number**
Breast	Invasive ductal	26
	Invasive lobular	2
Lung	Adenocarcinoma	9
	Squamous	8
	Small cell	3
Ovary	Adenocarcinoma	8
	Serous	5
	Endometroid	1
	Papillary	1
Colon	Adenocarcinoma	25
	Mucinous	1

**Table S2:** Correlation coefficients of suPAR values and *PLAUR* expression with various parameters

**Variable**		**Pearson’s rho**	**t (df)**	***p***
*suPAR*√**	Age	0.128	1.20 (87)	0.2
	BMI	−0.03	−0.35 (85)	0.72
	WBC	0.30	2.935 (86)	0.004*
	Fibrinogen	0.298	2.84 (83)	0.005*
	MPV/PLT ratio	−0.058	−0.51 (77)	0.61
*PLAUR mRNA log2(TPM+1)*	Age	0.124	6.32 (2529)	< 0.0001*
	*FGA*	0.299	15.91 (2564)	< 0.0001*
	*FGB*	0.152	7.8 (2564)	< 0.0001*
	*FGG*	0.295	15.17 (2398)	<0.0001*

suPAR: Soluble urokinase plasminogen activator; suPAR√: Square root transformed suPAR (ng/mL) to remove positive skewness; BMI: Body mass index; WBC: White blood cells; MPV/PLT: Mean platelet volume/platelet count; df: Degrees of freedom, *p-value < 0.01, TPM: Transcripts per million**.**
